# Neonatal exposure to high oxygen levels leads to impaired ischemia-induced neovascularization in adulthood

**DOI:** 10.1038/s41598-017-14396-8

**Published:** 2017-10-26

**Authors:** Raphael Mathieu, Sylvie Dussault, Michel Desjarlais, François Rivard, Wahiba Dhahri, Anik Cloutier, Anne-Monique Nuyt, Alain Rivard

**Affiliations:** 1 Department of Medicine, Centre Hospitalier de l’Université de Montréal (CHUM) Research Center, Montréal, Québec Canada; 2 Department of Pediatrics, Sainte-Justine University Hospital Research Center, Montréal, Québec Canada

## Abstract

Adverse perinatal conditions can lead to developmental programming of cardiovascular diseases. Prematurely born infants are often exposed to high oxygen levels, which in animal models has been associated with endothelial dysfunction, hypertension, and cardiac remodeling during adulthood. Here we found that adult mice that have been transiently exposed to O_2_ after birth show defective neovasculariation after hindlimb ischemia, as demonstrated by impaired blood flow recovery, reduced vascular density in ischemic muscles and increased tissue damages. Ischemic muscles isolated from mice exposed to O_2_ after birth exhibit increased oxidative stress levels and reduced expression of superoxide dismutase 1 (SOD1) and vascular endothelial growth factor (VEGF). Pro-angiogenic cells (PACs) have been shown to have an important role for postnatal neovascularisation. We found that neonatal exposure to O_2_ is associated with reduced number of PACs in adults. Moreover, the angiogenic activities of both PACs and mature mouse aortic endothelial cells (MAECs) are significantly impaired in mice exposed to hyperoxia after birth. Our results indicate that neonatal exposure to high oxygen levels leads to impaired ischemia-induced neovascularization during adulthood. The mechanism involves deleterious effects on oxidative stress levels and angiogenic signals in ischemic muscles, together with dysfunctional activities of PACs and mature endothelial cells.

## Introduction

Increasing evidence suggests that adverse perinatal events can induce developmental programming of future diseases during adulthood, particularly in the cardiovascular system^1,2^. For instance, a Swedish cohort study documented a 7% higher risk of dying from cardiovascular disease in young adulthood for each week of increased prematurity^3^. Preterm birth has also been associated with higher blood pressure, increased incidence of hypertension, and signs of vascular dysfunction in adolescents and young adults^2^. Infants born preterm are often exposed to high concentrations of O_2_, especially when compared to the relative hypoxic condition of the intrauterine life^4^. This can lead to increased oxidative stress^5^, a factor that is thought to be involved in several diseases of prematurity including retinopathy and bronchopulmonary dysplasia^6^. It has been suggested that exposure to high oxygen levels in infants born prematurely could also contribute to alter cardiovascular function during adulthood. In animal models, perinatal hyperoxia (a model of prematurity) has been associated with the adult development of endothelial dysfunction, hypertension, and cardiac remodeling^7,8^. However, the specific mechanisms that are involved in that pathophysiology are largely unknown. Moreover, how prematurity and transient neonatal exposure to high oxygen levels might modulate the physiological response to cardiovascular stresses in adults remains to be determined.

One of the most important adaptive mechanisms in the cardiovascular system is the response to ischemia and hypoxic stress. The capacity of the organism to counteract the negative effects of ischemia following vascular occlusion depends in large part on its ability to develop new vessels (neovascularization)^9^. Postnatal neovascularization is a complex phenomenon that necessitates the activation, proliferation and migration of mature endothelial cells (angiogenesis)^10^. Vascular endothelial growth factor (VEGF), an endothelial cell specific mitogen, has been shown to be a critical limiting factor for the induction of angiogenesis^11^. In addition, nitric oxide (NO) is recognized as an essential factor for endothelial function, VEGF-induced angiogenesis^12,13^, and ischemia-induced neovascularization^14^. Recently, it has been proposed that postnatal neovascularization does not only rely on the sprouting of pre-existing vessels, but also involves the contribution of bone marrow-derived pro-angiogenic cells (PACs)^15,16^. Evidence suggests that these cells are mobilized from the bone marrow into the peripheral blood in response to tissue ischemia. PACs migrate and reach sites of ischemia where they can promote neovascularization after differentiation into mature endothelial cells, or more often through paracrine secretion of growth factors and cytokines^17^.

Unfortunately, conditions leading to the development of atherosclerosis and vascular occlusions in patients are also often associated with impaired neovascularization in response to ischemia^9^. Cardiovascular risk factors such as aging, cigarette smoke exposure, diabetes and hypercholesterolemia have been associated with impaired ischemia-induced neovascularization and reduced number of PACs, both in animal models and in humans^9,17^. Preterm birth is increasingly recognized as a risk factor associated with the development of cardiovascular diseases later in life^1,2^. However, whether prematurity and/or perinatal adverse conditions can modify the physiological response to ischemia in adults is currently unknown. Here we used an animal model of prematurity and tested the hypothesis that neonatal exposure to hyperoxia might impair ischemia-induced neovascularization during adulthood. We also investigated potential mechanisms involved in that pathophysiology, including the effects of perinatal hyperoxia on the functional activities of mature endothelial cells and PACs.

## Results

### Effect of neonatal hyperoxia on blood flow recuperation after hindlimb ischemia

**Figure 1:**
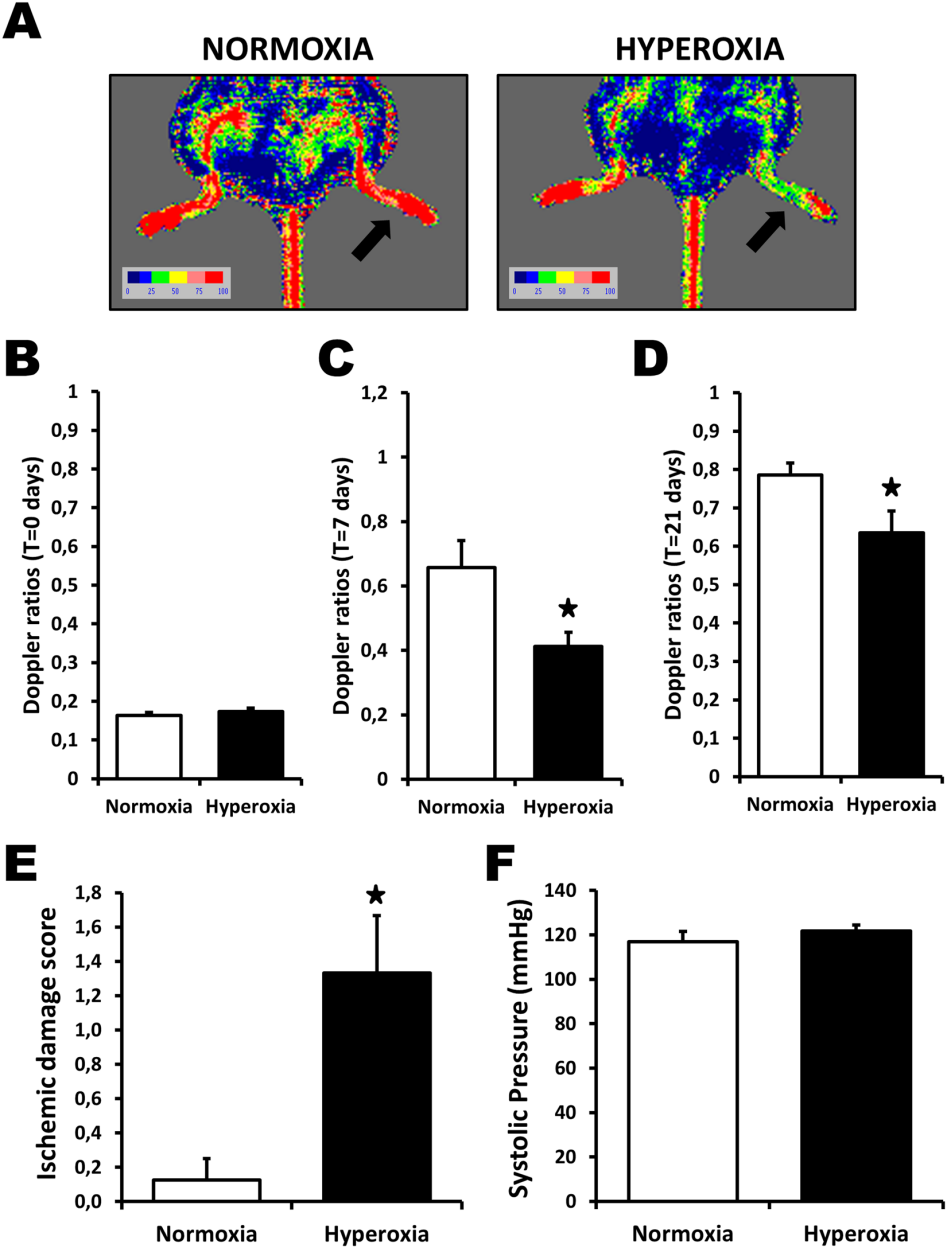
Neonatal hyperoxia and blood flow perfusion after hindlimb ischemia. (**A**) Pictures of representative Laser Doppler measurements 21 days after hindlimb ischemia. A color scale illustrates blood flow variation from minimal (dark blue) to maximal perfusion (red). Arrows indicate ischemic hindlimbs. (**B–D**) Laser Doppler perfusion ratios at baseline (**B**), day 7 (**C**) and day 21 (**D**) after ischemia. (**E**,**F**) Effect of neonatal hyperoxia on ischemic damages (**E**) and systolic blood pressure (**F**). Data are mean ± SEM (n = 7–12/group). *P < 0.05 vs. normoxia.

C57BL/6 mice were exposed to 85% O_2_ or room air (controls) from day 2 to 14 after birth. When the mice reached adulthood (8–10 weeks old), hindlimb ischemia was surgically induced by femoral artery removal. Compared to controls, mice exposed to O_2_ after birth showed a significant impairment of blood flow recuperation after hindlimb ischemia (Fig. 1A–D). Immediately after surgery (day 0), Laser Doppler flow ratios (DFR) between the ischemic and normal hindlimbs reached comparable low levels, indicating that the severity of the ischemia was similar in the 2 groups (Fig. 1B). However, DFR were significantly reduced in mice exposed to O_2_ after birth both at day 7 (DFR 0.41 ± 0.04 vs. 0.66 ± 0.08, *P* < 0.05, Fig. 1C) and at day 21 (DFR 0.63 ± 0.06 vs. 0.79 ± 0.03, *P* < 0.05, Fig. 1A and D). Clinically, this was also associated with increased hindlimb ischemic damages (Fig. 1E). Because neonatal exposure to high oxygen levels has previously been shown to increase blood pressure in rats, we measured blood pressure in adult mice that had been exposed or not to O_2_ after birth. As shown in Fig. 1F, blood pressure was similar in the 2 groups of mice, indicating that the impairment of blood flow recuperation in mice exposed to O_2_ after birth is not due to hypertension.

### Effect of neonatal hyperoxia in ischemic tissues

**Figure 2:**
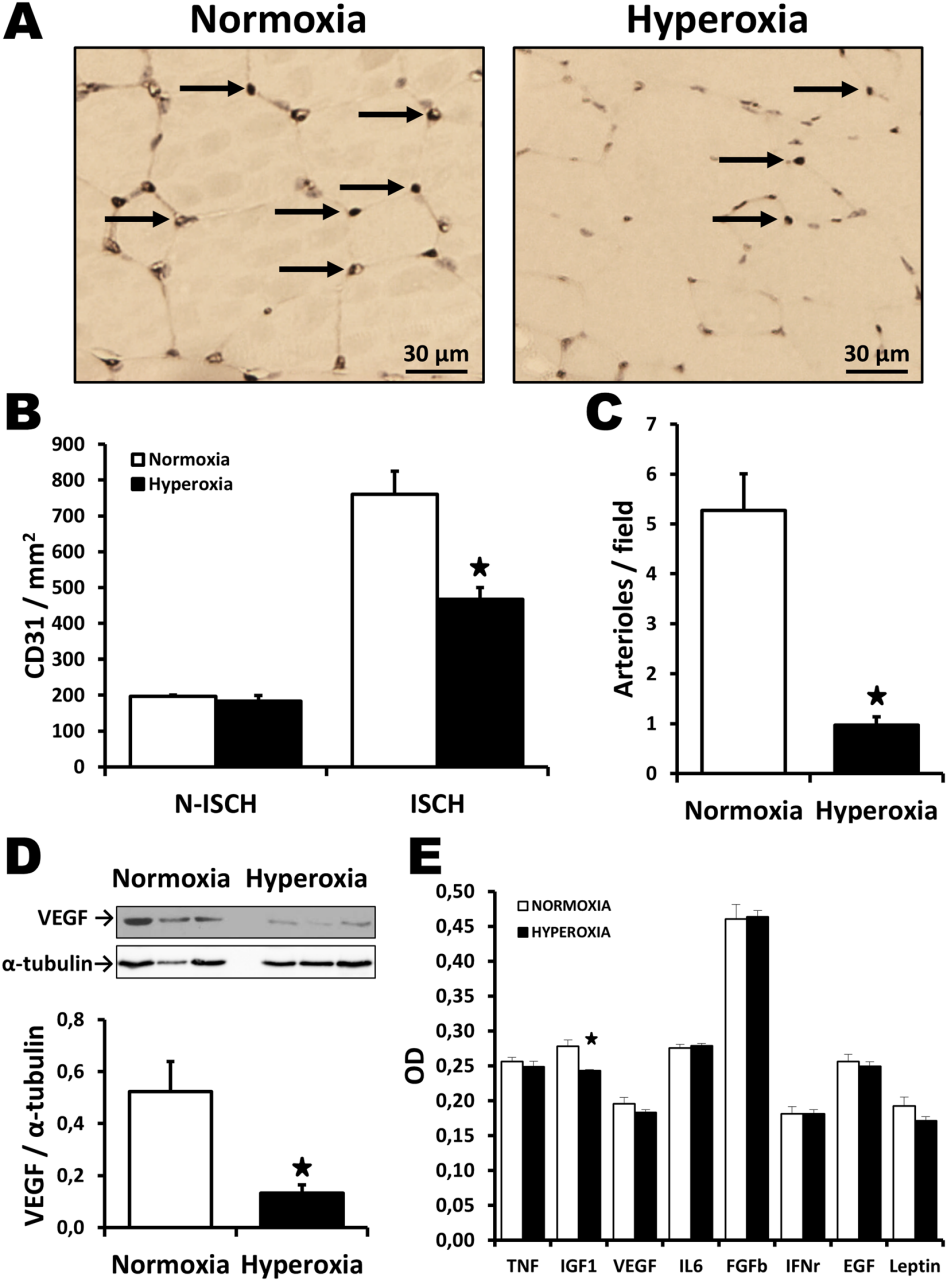
Effect of neonatal hyperoxia in ischemic tissues. Capillary density (**A**,**B**) and arteriolar density (**C**) in ischemic muscles harvested at day 21 after surgery in the different groups of mice. Arrows in (**A**) indicate positive (brown) CD31 staining in capillaries. Isch = ischemic. N-Isch = non ischemic. (**D**) Representative Western blots and quantitative analyses of VEGF expression in ischemic muscles at day 7 after surgery. Data were normalized using loading controls (alpha-tubulin) and are presented as mean ± SEM (n = 4/ group). (**E**) Effect of hyperoxia exposure on the level of angiogenic factors in the serum, as evaluated by Elisa at day 7 after surgery. *P < 0.05 vs. normoxia.

**Figure 3:**
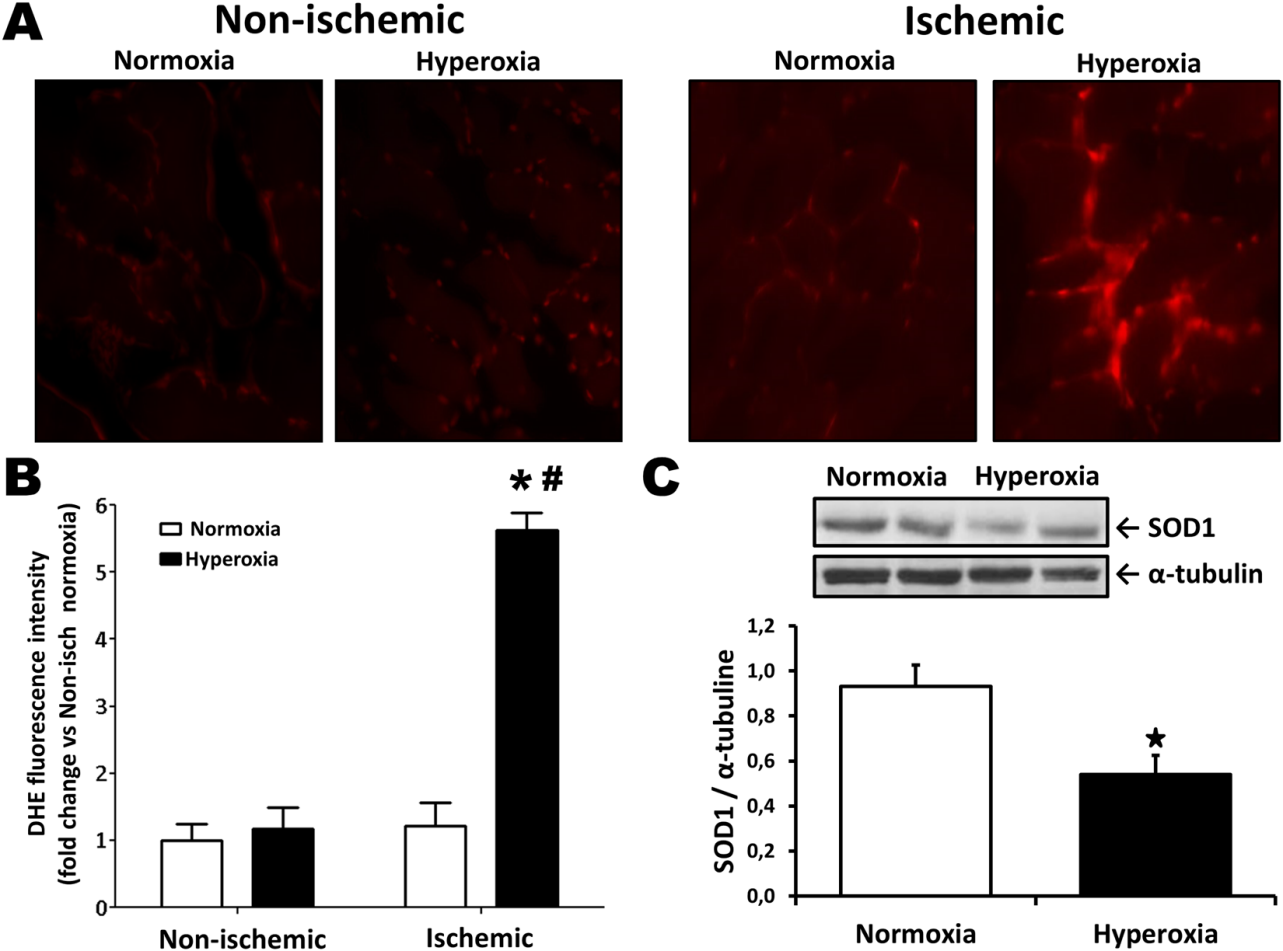
Neonatal hyperoxia and oxidative stress levels after ischemia. (**A**,**B**) Oxidative stress levels (DHE staining) in non-ischemic muscles and in ischemic muscles at day 7 after surgery. (**C**) Representative Western blots and quantitative analyses of SOD1 in ischemic muscles Data were normalized using loading controls (α-tubulin) and are presented as mean ± SEM (n = 8/group). *P < 0.05 vs. normoxia ischemic. ^#^ *P* < 0.05 vs. hyperoxia non-ischemic.

At the microvascular level, baseline capillary density was similar between groups in non-ischemic muscles (Fig. 2B). However, exposure to O_2_ after birth was associated with a significant reduction of capillary density (Fig. 2A,B **)** in ischemic hindlimb muscles at day 21 after surgery (467 ± 32 vs. 761 ± 64 capillaries per mm^2^, p < 0.05). Moreover, the number of arterioles (Fig. 2C) was also significantly decreased in mice exposed to O_2_ (1.0 ± 0._2_ vs. 5.3 ± 0.7 arterioles per field, p < 0.05). Interestingly, the expression of the angiogenic factor VEGF was found to be significantly reduced in the ischemic muscles of mice that had been exposed to O_2_ after birth (Fig. 2D **)**. VEGF levels were similar between groups in non-ischemic muscles (Supplemental Figure A and B). Systemically, there was a small reduction of IGF1 in the serum of mice that had been exposed to hyperoxia (Fig. 2E), but the levels of other angiogenic factors were similar between groups. Since excessive oxidative stress levels have been associated with impaired angiogenesis and reduced VEGF levels locally, we compared superoxide formation in the hindlimb muscles of mice exposed or not to high levels of O_2_ after birth. As shown in Fig. 3A,B, oxidative stress (DHE staining) was low in non-ischemic muscles. There was a small but non-significant increase of oxidative stress in ischemic muscles of mice exposed to normoxia. By contrast, mice exposed to hyperoxia exhibited an important increase of oxidative stress in ischemic muscles. Oxidative stress was localized in myocytes but also between muscle bundles in lectin positive vascular endothelial cells (data not shown). Ultimately, neonatal hyperoxia led to significant increase of superoxide levels in the ischemic muscles of adult mice. This was associated with a reduced expression of copper-zinc superoxide dismutase (CuZnSOD; SOD1), a major antioxidant enzyme involved in the modulation of vascular health and angiogenesis (Fig. 3C). SOD1 levels were similar between groups in non-ischemic muscles (Supplemental Figure A and C).

### Effects of neonatal hyperoxia on mature endothelial cells *in vitro*

**Figure 4:**
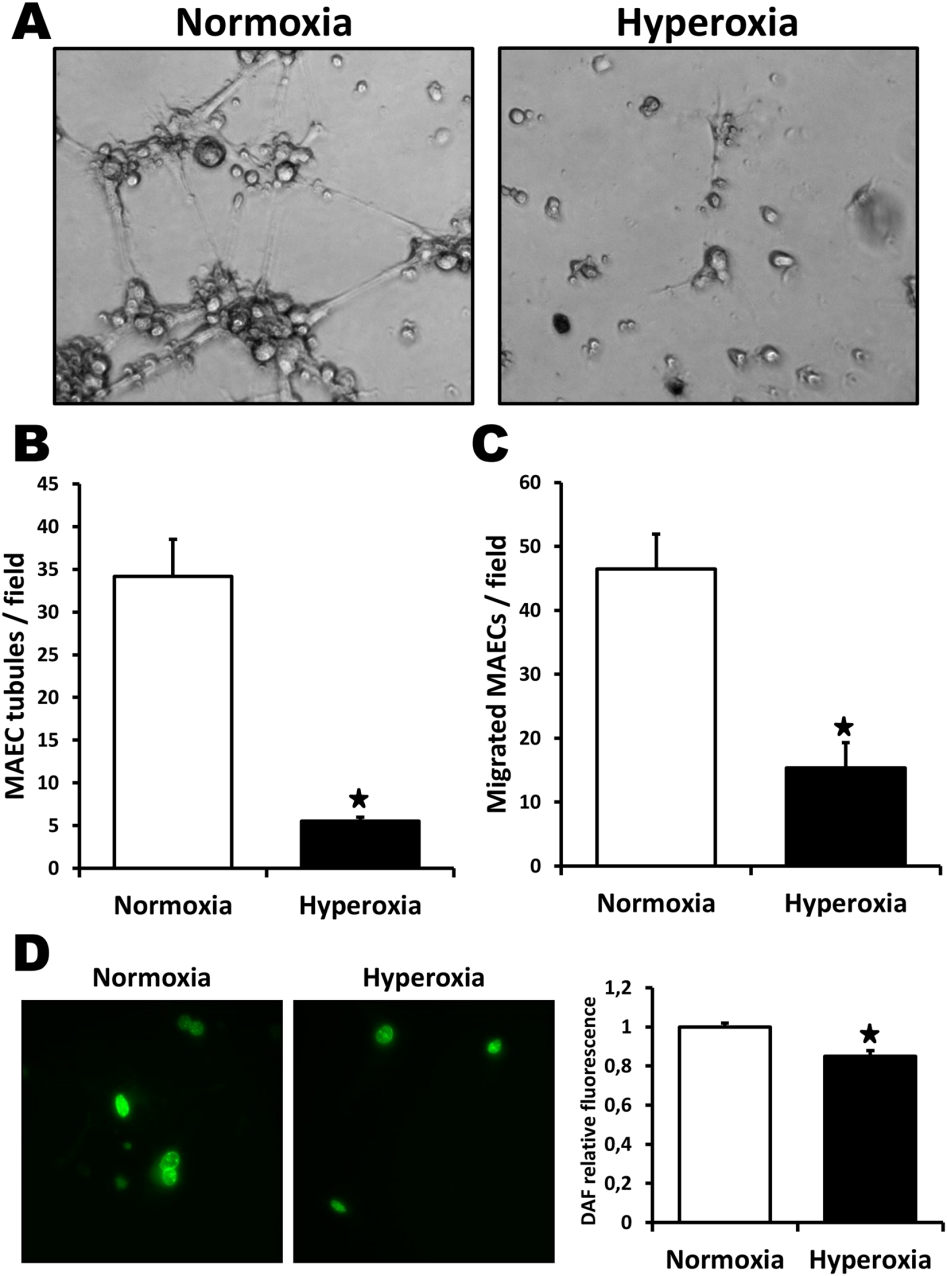
Effect of neonatal hyperoxia on mature endothelial cells *in vitro*. Analyses of tube formation (**A**,**B**), cellular migration (**C**) and nitric oxide formation (**D**) in MAECs isolated from adult mice that have been exposed to neonatal hyperoxia or normoxia. Data are presented as mean ± SEM. *P < 0.05 vs. normoxia.

To better characterize the effects of neonatal hyperoxia on angiogenesis, *in vitro* studies were performed using mouse aortic endothelial cells (MAECs) isolated from adult mice. As shown in Fig. 4, neonatal hyperoxia was associated with endothelial dysfunction during adulthood. MAECs isolated from adult mice exposed to high levels of O_2_ after birth exhibited impaired tubule formation (Fig. 4A,B) and cellular migration (Fig. 4C **)** compared to MAECs isolated from control mice. Moreover, NO expression (DAF staining) was significantly reduced in MAECs originating from O_2_–exposed mice (Fig. 4D).

### Effect of neonatal hyperoxia on PAC number and function

**Figure 5:**
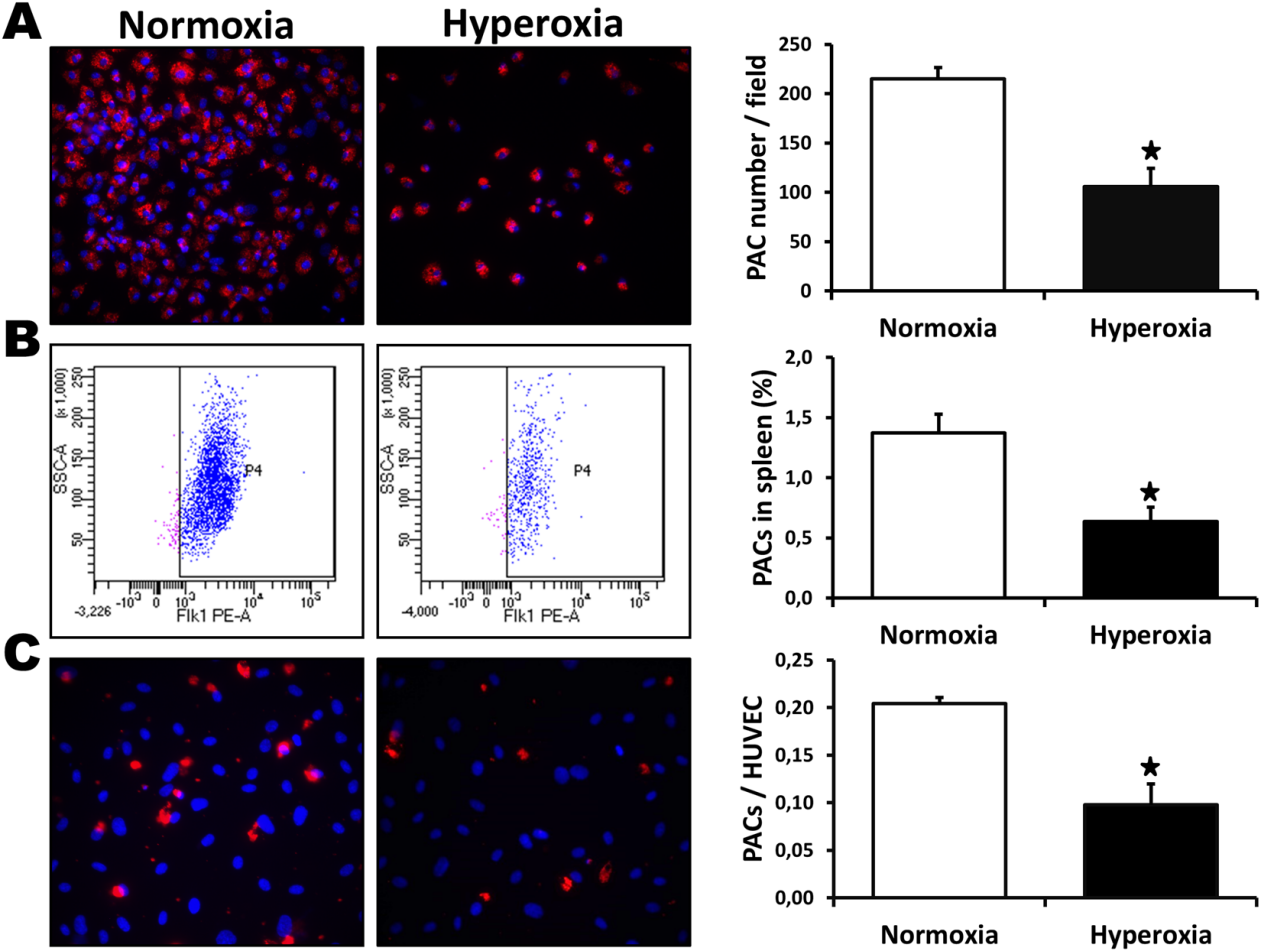
Effect of neonatal hyperoxia on PAC number and function. Number of PACs isolated from the bone marrow (**A**) and % of PACs in the spleen as assessed by flow cytometry (**B**) in mice exposed to neonatal hyperoxia or normoxia. To assess PAC adhesion (**C**), PACs were labelled with a DiI fluorescent marker and allowed to adhere to a monolayer of tumor necrosis factor-α-stimulated HUVECs. Data are mean ± SEM (n = 4–6/group). *P < 0.05 vs. normoxia.

PACs have been shown to reach sites of neovascularization where they can contribute to the formation of new blood vessels. We investigated the effect of neonatal hyperoxia on the number and the functional activities of PACs in adult mice. The number of PACs in the bone marrow of mice that had been exposed to high levels of O_2_ after birth was significantly reduced compared to control mice (Fig. 5A). Similarly, the percentage of PACs contained in the total viable cell population derived from the spleen (FACS analysis) was also significantly reduced in mice exposed to O_2_ after birth (Fig. 5B). In addition, we observed that PACs isolated from mice exposed to O_2_ after birth showed decreased adhesion to endothelial cells (Fig. 5C) compared to PACs isolated from control mice.

## Discussion

To our knowledge, the present study is the first documentation of the negative effect of neonatal hyperoxia on ischemia-induced neovascularization during adulthood. We used an established mouse model of prematurity, where pups were exposed to hyperoxic conditions (85% O_2_) from day 2 to 14 after birth^18^. Since organ maturation continues after birth in rodents, the immediate neonatal period in these animals is thought to reflect a developmental time equivalent in humans to the last trimester of gestation. Hyperoxia in this model replicates the ex utero marked rise in PO_2_ (blood partial pressure in oxygen) occurring at delivery, a phenomenon that is accentuated in premature infants treated with high levels of oxygen. Even short-term (2–5 hours) exposure to hyperoxia has been associated with organ dysfunction after ischemia in some models^19^. Neonatal exposure to hyperoxia in rodents is a recognized model to study classical (lungs, eyes) O_2_-related complications of prematurity^20,21^. Moreover this model has also been used to document developmental programming of future diseases in the cardiovascular system, including hypertension^7^ and cardiac dysfunction^8^. Our study extends these previous findings by showing that neonatal exposure to hyperoxia also significantly impairs the future physiological response to ischemia (i.e. neovascularization) in adults. This could have important clinical implications since prematurity has been associated with a higher risk of dying from cardiovascular disease in young adulthood^3^. Therefore improving neovascularization and blood flow recovery in the context of tissue ischemia could constitute an important therapeutic target in this population.

We used a well-characterized mouse model of hindlimb ischemia^22^ to define the effects of neonatal hyperoxia on post-natal neovascularization in adults. We found that previous neonatal exposure to high levels of O_2_ was associated with a significant impairment of neovascularization following hindlimb ischemia in adult animals. This was reflected by a reduced blood flow recovery after surgery, together with decreased vascular densities (both capillaries and arterioles) in ischemic muscles. Clinically, the reduction in neovascularization was also associated with increased hindlimb ischemic damages. The mechanisms involved in the inhibition of ischemia-induced neovascularization are potentially diverse. Interestingly, neonatal hyperoxia has previously been associated with increased blood pressure levels in adult rats whereas both angiogenesis^23,24^ and vasculogenesis (EPC function)^25^ have been shown to be impaired in animal models of hypertension. In humans, hypertension is more prevalent among adults born preterm^2^ and preterm-born individuals exhibit an enhanced antiangiogenic state in adult life that is specifically related to elevations in blood pressure^26^. However, here we found that blood pressure levels were not significantly increased in mice that had been exposed to hyperoxia after birth. Compared to previous findings, this might reflects differences in the animal model used (mice vs. rats) and/or be related to the fact that animals were studied at a relatively young age (8–10 weeks old) in the current study. In any case, our data suggest that the deleterious effect of neonatal hyperoxia on ischemia-induced neovascularization in adults is not due to hypertension.

An important factor that could be involved in the modulation of neovascularization is oxidative stress. Infants born prematurely have reduced antioxidant defenses^27^ and are often exposed upon birth to high concentrations of O_2_ and pro-oxidant conditions^6^. This oxidative injury is thought to be an important factor involved in short-term complications of prematurity such as bronchopulmonary dysplasia and retinopathy of prematurity^28^. It has also been associated with reduced pulmonary angiogenesis in neonates^29^. Interestingly, our results indicate that adult mice that have been exposed to hyperoxia after birth also exhibit increased oxidative stress in ischemic muscles compared to control mice (Fig. 3). Angiogenesis and neovascularization have been shown to be impaired in conditions where oxidative stress levels are increased, a situation that can be improved by antioxidant therapies or genetic manipulations^30–32^. Moreover, reactive oxygen species (ROS) can impair the activity of VEGF^30,33^, a critical limiting factor involved in the initiation and the maintenance of angiogenesis^11^. Here we found that increased oxidative stress levels in ischemic muscles of mice exposed to hyperoxia after birth was associated with a significant reduction of VEGF expression (Fig. 2D). It is possible that impaired defense against oxidative stress persists during adulthood in individuals that have been exposed to hyperoxia after birth. Consistent with this, we found that the antioxidant enzyme CuZnSOD (SOD1) was significantly reduced in the ischemic muscles of mice that had been exposed to hyperoxia after birth compared to control mice. The functional importance of SOD1 has been demonstrated in homozygous SOD1-deficient mice, which present increased superoxide levels and altered responsiveness in both large arteries and microvessels^34^. Moreover, we have previously shown that increased oxidative stress levels in SOD1-deficient mice lead to impaired ischemia-induced neovascularization^35^ and accelerated vascular aging^36^. Therefore, combined with previous studies, our results suggest that defective antioxidant mechanisms (including SOD1) could contribute to impair VEGF activity and ischemia-induced neovascularization in adult mice exposed to neonatal hyperoxia. However the mechanisms involved in the developmental programming of defective antioxidant defense in adults, especially in the context of tissue ischemia, remain to be defined.

The results of the present study suggest that PACs could also be involved in the inhibition of neovascularization in adults that have been exposed to hyperoxia after birth. PACs are recruited into ischemic tissues where they can facilitate neovascularization directly through incorporation into new vessels, or indirectly by secreting angiogenic factors in a paracrine fashion^17^. The effect of prematurity on the number of PACs is unclear. Previous studies have found increased, reduced or equal number of PACs in preterm infants compared to term controls^37^. However, preterm PACs seem more vulnerable to exogenous factors such as oxidative stress^37^. To our knowledge, no study has examined the effects of prematurity and/or neonatal hyperoxia on PAC number and function beyond the neonatal period. Here we found that previous neonatal exposure to hyperoxia is associated in adult animals not only with defective angiogenic activities of mature endothelial cells, but also with a significant reduction in the number and the functional activities of PACs. Although the mechanisms involved in that physiopathology are currently unknown, the deleterious effects of neonatal hyperoxia on PACs could be at least in part related to increased ROS levels. Indeed, previous reports have shown that excessive oxidative stress leads to impaired PAC angiogenic activities^35,38^. In future studies, it will be interesting to determine whether defective antioxidant defense mechanisms are found in PACs isolated from adults born preterm, which could contribute to impair PAC function and neovascularization in these individuals.

In conclusion, our study demonstrates for the first time that transient neonatal exposure to high oxygen levels leads to impaired ischemia-induced neovascularization during adulthood. We propose that this occurs through deleterious effects on oxidative stress levels and angiogenic signals in ischemic muscles, together with dysfunctional activities of mature endothelial cells and PACs. Our study sheds light on a novel mechanism that could contribute to increase the incidence and morbidity of cardiovascular diseases in individuals born preterm. The development of strategies to therapeutically improve neovascularization in this population could have important clinical implications.

## Methods

### Animals and exposure to hyperoxia

The animal protocol was approved and all experiments were conducted in accordance with the Comité Institutionnel de Protection des Animaux (CIPA) of the Centre Hospitalier de l’Université de Montréal (CHUM). Time-dated pregnant female C57Bl/6 mice were obtained from Charles River (St-Constant, Canada), maintained in a 12 h light-dark cycle and fed ad libitum. At day 2 after birth, pups were divided in two groups: the control group was maintained in normoxic conditions whereas the second group was exposed to hyperoxic conditions (85% O_2_ by a mixture of medical grade 100% O_2_ and room air with an oxycycler ProOx model 110, Biospherix, Lacona, NY) as previously described^18,39^. Nursing dams were rotated between the hyperoxia and normoxia groups every 24 h to avoid maternal oxygen toxicity. Fourteen days after birth, exposure to hyperoxia was ended and all mice were then maintained in normoxic room air until the end of the study. For the measurement of angiogenic growth factors in the serum, enzyme-linked immunosorbent assays (ELISA) were performed using the mouse angiogenesis ELISA strip assays from Signosis (Sunnyvale, CA) according to the manufacturer’s instructions.

### Murine ischemic hindlimb model and blood flow monitoring

When mice reached 8–10 weeks of age, unilateral hindlimb ischemia was surgically induced after anesthesia with 2% isoflurane as previously described^40^. Our model does not involve necrosis and tissue repair in hindlimb muscles but can be associated with ischemic damages distally in the foot and toes. Hindlimb blood flow was assessed at day 21 after surgery using a Laser Doppler perfusion imager (LDPI) system (Moor Instrument Ltd., Axminster, UK)^40^. Low and/or no perfusion was displayed in dark blue, whereas the highest perfusion interval was displayed in red. To account for variables such as ambient light and temperature, the results are expressed as the ratio of perfusion in the left (ischemic) vs. right (non-ischemic) hindlimb. Ischemic damage of foot and toes was evaluated using a scale from 0 (no necrosis) to 4 (amputation). Blood pressure was measured using a tail-cuff pressure instrument (BP-2000, Visitech Systems, Apex, NC)^41^.

### Tissue preparation and immunochemistry

Whole ischemic hindlimb muscles were immediately fixed in tissue-fix overnight. Identification of endothelial cells was performed by immunostaining for mouse platelet endothelial cells adhesion molecule-1 (PECAM-1 or CD31) with a rat monoclonal antibody (Pharmingen, San Diego, CA). Capillaries, identified by positive staining of CD31 and appropriate morphology, were counted by a single observer blinded to the treatment regimen under a 200x magnification to determine the capillary density. Serial sections were cut at three different levels, and representative fields were analyzed by counting the number of capillaries in each field. Arterioles were identified using a Modified Verhoeff Van Gieson Elastic Stain Kit (Sigma, St- Louis, MO). Serial sections were cut at three different levels, and positive vessels identified by the presence of a continuous internal elastic laminae were analyzed for the entire section under a 100x magnification^41^.

### Detection of superoxide in ischemic muscle

To evaluate superoxide production in ischemic muscles, dihydroethidium (DHE) fluorescence labeling was performed^41^. After bones had been carefully removed, ischemic muscles were put in a 25% sucrose solution for 10 minutes and were frozen in eppendorf tubes at −80 °C for 24 h, molded in OCT and kept at −20 °C. 3 mm frozen sections were made at three different levels in the ischemic muscles. Sections were labeled with 10 mM DHE (Calbiochem, San Diego, CA) for 30 minutes. Intensities of fluorescence were measured and analyzed using computer-based software (ImageJ) with the same threshold for all sections under a 100x magnification. The specificity of the test was confirmed by pre-incubating the section with superoxide dismutase polyethyleneglycol (PEG-SOD) 500 U/ml for 1 h (data not shown).

### Western blot analysis

Hindlimb muscles were rinsed in PBS, frozen in liquid nitrogen and stored at −80 °C. Protein extracts were obtained after homogenization of muscles in ice-cold RIPA lysis buffer. The membranes were probed with the following antibodies: 1:1000 VEGF (Santa Cruz Biotechnology, Dallas, TX), 1:1000 SOD-1 (Cell Signaling Technology, Danvers, MA) and 1:5000 alpha-tubulin (Abcam, Toronto, Canada). Protein expression was quantified by high-resolution optical densitometry (image J software). Results are expressed as density values normalized to the loading control.

### Flow cytometry analysis of pro-angiogenic cells (PACs)

The percentages of PACs contained in the total viable cell population derived from the spleen was measured by flow cytometry (FACSCalibur flow cytometer, Becton Dickinson, Oakville, Ontario, Canada) using the fluorescence-coupled cell markers CD34-FITC, VEGFR-2 (Flk1)-PE and CD117 (c-kit)-APC (eBioscience, San Diego, CA). Cell phenotypes were determined by the analysis of 300 000 events^41^.

### PACs isolation and characterization

Seven days after hindlimb ischemia, mouse bone marrow mononuclear cells were isolated from the femora, tibiae and humeri bones by flushing the bone marrow cavities using culture medium (medium 200 (Life Technologies) supplemented with 18% fetal bovine serum (FBS, Life Technologies) and low serum growth supplement (2% FBS, 3 ng/ml bFGF, 10 mg/ml heparin, 1 mg/ml hydrocortisone, and 10 ng/ml EGF; Life Technologies)). After red blood cell lysis and washing, bone marrow mononuclear cells were plated on 0.005% fibronectin (Sigma). After 4 days in culture, non-adherent cells were removed by thorough washing with PBS. Adherent cells were stained with 1,10-dictadecyl-3,3,30,30 tetramethyllindocarbocyanine perchlorate-acetylated low-density lipoprotein (DiI-acLDL, 2.5 mg/ml for 1 h; Alfa Aesar, Tewksbury, MA). Bone marrow PACs were characterized as adherent cells that were positive for DiI-acLDL uptake^41^.

### PAC adhesion to an endothelial monolayer

A monolayer of confluent human umbilical vein endothelial cells (HUVECs; passage 3–5) was prepared in 24 well plates. HUVECs were pretreated for 16 h with tumor necrosis factor-α (1 ng/ml; BD Biosciences, Mississauga, Canada), fixed and stained with DAPI (0.5 mg/ml; Life Technologies). PACs were labeled with DiI-AcLDL and 15 000 PACs were added to each well (2 wells/mouse) and incubated for 3 h at 37 °C. Non-attached cells were gently removed by PBS washing and adherent PACs were fixed with 2% paraformaldehyde and counted in three random fields per well^42^.

### Isolation and culture of aortic endothelial cells

Endothelial cells were isolated from the thoracic aorta using an explant technique^32^. The thoracic aorta was opened longitudinally and cut into 2 mm-long explants. The aortic segments were placed face-down on a Matrigel-coated 6-well plates (2 aorta per well; Basement membrane, BD Biosciences) and incubated in DMEM supplemented with 10% FBS, 10% Newborn Calf serum, 1% penicillin-streptomycin (Life Technologies), 90 ug/mL heparin (Sigma), 50 ug/ml endothelial cell growth supplements (VWR), and 100 U/mL fungizone (Life Technologies) at 37 °C in a 95% air/5% CO_2_ incubator. The vessel segments were removed once cell outgrowth was observed (around day 7). 1–2 weeks later, the cells were detached with dispase (BD Biosciences) and plated onto 1% gelatin–coated 25 cm^2^ flasks. Cells were used at early passage (P2).

### Capillary-like tube formation on Matrigel

Endothelial cells were plated (20 000) in 96-well plates that had been pre-coated with 50 μl of growth factor reduced Matrigel Matrix (BD Biosciences) and cultured at 37 °C for 6 h with 50 ng/ml of VEGF. Capillary-like tubes were then photographed under an inverted microscope and all side branches were counted by a single investigator in a blinded manner^42^.

### Cell migration assay

Cell migration was assessed using a modified Boyden chamber assay. Polyvinylpyrrolidine-free polycarbonate filter Transwell inserts (6.4 mm diameter, 8 μm pores; Costar, Cambridge, MA) were coated with 0.1% gelatin. Inserts were placed in a 24-well plate containing medium with 50 ng/ml of VEGF. Cells were allowed to migrate from the upper to the lower chamber for 6 h at 37 °C. The number of cells that had migrated was counted in three different representative high power (200x) fields per insert (2 inserts/condition)^42^.

### Detection of intracellular nitric oxide (NO)

Intracellular generation of NO was visualized with diaminofluorescein-2-diacetate (DAF-2DA, Cell Technology). Cells were incubated with DAF-2DA for 30 minutes. Fluorescence intensities were quantified using imageJ, with the same threshold for each experiment^41^.

### Statistical analysis

All results are presented as mean ± SEM. Statistical significance was evaluated by unpaired t-test. A value of P < 0.05 was interpreted to denote statistical significance.

### Data availability

All data generated or analyzed during this study are included in this published article.

## Electronic supplementary material

Supplemental Figure
