# Supplementary material for: Neonatal exposure to high oxygen levels leads to impaired ischemia-induced neovascularization in adulthood
Source: Sci Rep. 2017 Oct 26;7:14143. doi: 10.1038/s41598-017-14396-8 (PMC5658429; doi:10.1038/s41598-017-14396-8)
Supplement: Supplementary file 1 — Supplemental Figure [file 41598_2017_14396_MOESM1_ESM.pdf]

Neonatal exposure to high oxygen levels leads to impaired ischemia-induced neovascularization in adulthood

Raphael Mathieu, Sylvie Dussault, Michel Desjarlais, François Rivard, Wahiba Dhahri, Anik Cloutier, Anne-Monique Nuyt and Alain Rivard

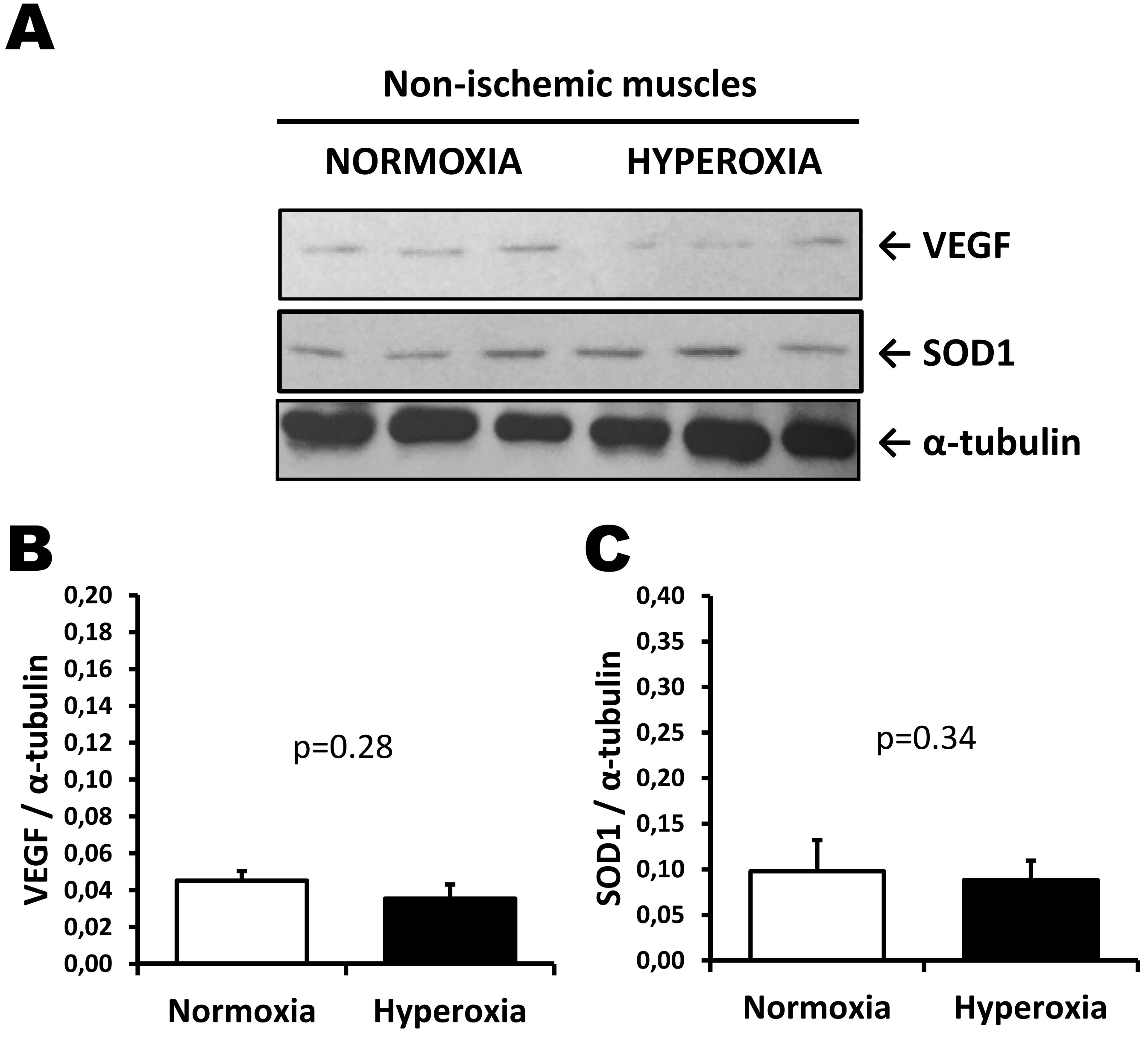

Supplemental Figure: Effect of perinatal exposure to hyperoxia on VEGF and SOD1 expression in non-ischemic hindlimb muscles. Representative Western blots (**A**) and quantification of VEGF (**B**) and SOD1 (**C**) expression in the different conditions. p=ns for both VEGF (p=0.28) and SOD1 (p=0.34).
